# Faster Cognitive and Functional Decline in Dysexecutive versus Amnestic Alzheimer's Subgroups: A Longitudinal Analysis of the National Alzheimer's Coordinating Center (NACC) Database

**DOI:** 10.1371/journal.pone.0065246

**Published:** 2013-06-03

**Authors:** Jesse Mez, Stephanie Cosentino, Adam M. Brickman, Edward D. Huey, Jennifer J. Manly, Richard Mayeux

**Affiliations:** 1 Gertrude H. Sergievsky Center, College of Physicians and Surgeons, Columbia University, New York, New York, United States of America; 2 Taub Institute for Research on Alzheimer’s Disease and the Aging Brain, College of Physicians and Surgeons, Columbia University, New York, New York, United States of America; 3 Department of Neurology, College of Physicians and Surgeons, Columbia University, New York, New York, United States of America; 4 Department of Biostatistics, Mailman School of Public Health, Columbia University, New York, New York, United States of America; 5 Department of Psychiatry, College of Physicians and Surgeons, Columbia University, New York, New York, United States of America; 6 Department of Epidemiology, Mailman School of Public Health, Columbia University, New York, New York, United States of America; University Of São Paulo, Brazil

## Abstract

**Objective:**

To compare the rate of cognitive and functional decline in dysexecutive, typical and amnestic subgroups of Alzheimer’s disease.

**Methods:**

943 participants from the National Alzheimer’s Coordinating Center (NACC) database who had a diagnosis of probable AD were followed for a mean of 2.3 years. A dysexecutive subgroup (n = 165) was defined as having executive performance >1.5 SD worse than memory performance, an amnestic subgroup (n = 157) was defined as having memory performance >1.5 SD worse than executive performance and a typical subgroup (n = 621) was defined as having a difference in executive and memory performance of <1.5 SD. Generalized estimating equations (GEE) were used to model decline on the Folstein Mini Mental Status Exam (MMSE), rise on the Clinical Dementia Rating (CDR) sum of boxes and rise on the total Functional Assessment Questionnaire (FAQ).

**Results:**

Compared with the amnestic subgroup, the dysexecutive subgroup declined 2.2X faster on the Folstein MMSE (p<.001), rose 42% faster on the CDR sum of boxes (p = .03) and rose 33% faster on the total FAQ (p = .01). Rate of change for the typical subgroup fell between that of the amnestic and dysexecutive subgroups for the MMSE, CDR sum of boxes and total FAQ. Among a subset of participants (n = 129) who underwent autopsy, the dysexecutive, amnestic and typical subgroups did not differ in odds of having an AD pathologic diagnosis, suggesting that variation in non-AD pathologies across subtypes did not lead to the observed differences.

**Conclusions:**

A dysexecutive subgroup of AD has a unique disease course in addition to cognitive phenotype.

## Introduction

While Alzheimer’s disease (AD) “classically” presents with predominant episodic memory deficits [1], in actuality, the presentation can be quite heterogeneous. Examples of atypical presentations include logopenic primary progressive aphasia, posterior cortical atrophy/visual variant, and a dysexecutive variant [2], [3]. Executive dysfunction refers to deficits in “planning, judgment, reasoning, problem solving, organization, attention, abstraction and mental flexibility” [4]. A subset of mild AD patients (clinical dementia rating (CDR) [5] of 0.5 or 1) has been described with substantial executive dysfunction relative to dysfunction in other cognitive domains on neuropsychological testing [3].

We and others have shown that this dysexecutive subgroup of AD has unique genetic, biological, and clinical characteristics compared with typical AD. One study found that the dysexecutive subgroup has disproportionate amyloid plaque burden in the frontal lobes [6], while another found disproportionate amyloid plaque and neurofibrillary tangle burden in the frontal lobes as compared to the typical distribution of pathology in AD [7]. Structural and functional imaging studies suggest that AD patients with predominant executive dysfunction have greater frontoparietal cortical thinning and hypometabolism than healthy controls as well as AD patients with predominant memory deficits [8], [9]. We and others have found that the *APOEε4* allele is less frequent in a dysexecutive subgroup of AD compared with an amnestic subgroup [9]–[11]. We have also shown that hypertension is less frequent in a dysexecutive AD subgroup compared with an amnestic AD subgroup [11].

The literature shows that AD patients with poor executive function tend to have more difficulty with activities of daily living even when controlling for global cognition and memory [12]–[15]. There is also a suggestion that these patients tend to have a faster decline in daily function [9], though a thorough investigation has not been completed. Further, it is unknown whether AD patients with predominant executive dysfunction versus memory dysfunction show different rates of cognitive decline. Here we address these issues further.

## Methods

### Ethics Statement

The National Alzheimer's Coordinating Center (NACC) developed and maintains a large relational database of standardized clinical research data collected from the NIA-funded Alzheimer's disease Centers (ADCs) nationwide [16]. Data collection was approved by an institutional review board at each ADC. The active ADCs include Arizona Alzheimer’s Center, Boston University, Columbia University, Duke University Medical Center, Emory University, Indiana University, Johns Hopkins University, Massachusetts ADRC, Mayo Clinic, Mount Sinai School of Medicine, New York University, Northwestern University, Oregon Health and Science University, Rush University Medical Center, University of California – Davis, University of California – Irvine, University of California – Los Angeles, University of California – San Diego, University of California – San Francisco, University of Kansas, University of Kentucky, University of Pennsylvania, University of Pittsburgh, University of Southern California, University of Texas Southwestern, University of Washington, University of Wisconsin and Washington University. Each ADC has an individual mechanism for obtaining informed consent from participants or, if they lacked capacity to consent, from next of kin, care takers or guardians. On submission to the NACC database, data were de-identified. All analyses for the current study were performed anonymously.

The current study is a secondary analysis of NACC data collected between 2005 and 2011. Recruitment, participant evaluation, and criteria for dementia and probable AD are detailed elsewhere [17]. Participants were followed at approximately 12 month intervals with similar evaluations and reassessment of the diagnosis at each timepoint. Participants were either prevalent cases (i.e. were given an AD diagnosis at initial visit) or incident cases (i.e. were given an AD diagnosis at a follow-up visit). For incident cases, visits prior to the AD diagnosis were not included in this analysis and future mention of ‘baseline’ visit refers to initial visit at which an AD diagnosis was made. Stratification by incident/prevalent status yielded similar effect sizes and thus incident and prevalent cases were combined in the analysis. Because we were interested in the early presentation of AD, we restricted our sample to participants who met criteria for probable mild AD (CDR≤1) at baseline visit.

Race (white, African American, American Indian or Alaska native, Pacific Islander, Asian or other) and presence of Hispanic/Latino ethnicity were ascertained by self-report using two separate questions. All future references to African Americans and whites imply non-Hispanic African Americans and non-Hispanic whites. Achieved years of education were ascertained by self-report.

A subset of participants was administered the ADCs’ Uniform Data Set (UDS) neuropsychological battery at the baseline visit. The current study uses the following tests from the UDS: Trail Making Test (TMT) part A, TMT part B, Logical Memory Test story A (LMTA) immediate recall and LMTA delayed recall [18]. The Folstein Mini Mental Status Exam (MMSE) was administered at each visit [19].

Daily function was assessed using the Clinical Dementia Rating (CDR). Box scores in memory, orientation, judgment and problem solving, community affairs, home and hobbies, and personal care were assigned according to CDR scoring criteria. A standard global CDR and standard CDR sum of boxes were calculated from the box scores [5]. While every participant was assigned a global CDR at each visit, CDR sum of boxes and CDR box scores were only recorded for a subset of participant visits. All participant visits with a recorded CDR sum of boxes had complete individual box scores. Functional status was also assessed at each evaluation using the Functional Assessment Questionnaire (FAQ). On the FAQ, functional status is divided into 10 different categories. For each category, a score of 0–3 corresponds to “normal,” “has difficulty, but does by self,” “requires assistance” or “dependent” respectively. A total functional score was calculated by summing the category scores. A higher score indicates more functional impairment [20].

*APOE* genotype was determined for a subset of participants. They were classified as having no *APOEε4* allele, one *APOEε4* allele or two *APOEε4* alleles.

943 (21.0%) of the 4,491 eligible participants were included in the study. Participants were excluded if they did not or were too impaired to complete neuropsychological testing at the baseline visit. For Trails testing, participants were not included if they were at the floor (150s for Trails A, 300s for Trails B) or could not complete both Trails A and Trails B. Participants were included and assigned a Trails B time of 300s if they were above the floor for Trails A, but were at the floor or could not complete Trails B. This served as a compromise between excluding participants who may have had an extreme dysexecutive phenotype and excluding participants who were too impaired to be meaningfully assigned to a subgroup. Participants were also excluded if they lacked *APOE* genotyping or did not have a follow-up visit. Included and excluded participants did not differ in age. Compared with included participants, excluded participants had greater odds of identifying as African American (OR = 2.1, p<.001), had greater odds of being female (OR = 1.4, p<.001) and had 0.6 fewer years of education (p<.001).

129 of the eligible participants, who had neuropsychological testing, underwent autopsy. Compared with the non-autopsy subset, the autopsy subset had lower odds of identifying as African American (OR = 0.33, p = .02), had lower odds of being female (OR = 0.47, p<.001), was 3.9 years older (p<.001), and had 1.0 more years of education. Each autopsy participant was given a primary pathologic diagnosis.

Classification of the ‘dysexecutive’, ‘amnestic’ and ‘typical’ subgroups was made at the baseline visit according to methods used previously [11], but nonetheless is detailed below for completeness. The LMTA and the TMT were used to evaluate memory and executive function respectively. This method was chosen because it utilizes neuropsychological tests that are widely used so it can be easily replicated. Further, the method has been shown to differentiate patients into subgroups who demonstrate consistent generalizable deficits in their respective cognitive domains on multiple neuropsychological tests [9], [11]. In the LMTA, delayed recall was subtracted from immediate recall to account for learning ability. This value was termed the memory score. Lower scores reflect better retention of information over time. In the TMT, TMT A was subtracted from TMT B to account for attention, processing speed and basic visuo-motor abilities. Lower scores are indicative of better performance (i.e. less difficulty performing the executive component of the task). This value was termed the executive score. A mean and standard deviation for the executive and memory scores were calculated. These were used to calculate Z scores for each participant. Participants were classified as dysexecutive if their executive performance was ≥1.5 SD below their memory performance. Participants were classified as amnestic if their memory performance was ≥1.5 SD below their executive performance. Participants were classified as typical if their memory and executive scores differed by <1.5 SD. We chose to call this subgroup ‘typical’ because the majority of cases fell into this phenotypic subgroup, reflecting the fact that AD patients generally present with both memory and executive deficits.

### Statistical Analysis

Among the pathologic subset, a Pearson Chi Squared test was used to compare the odds of an AD pathologic diagnosis in the dysexecutive, amnestic and typical subgroups.

In the full sample, a one-way ANOVA was used to compare age at baseline, years of education, years followed, years from reported symptom onset to the baseline visit, number of visits, baseline MMSE, baseline CDR sum of boxes and baseline total FAQ in the dysexecutive, typical and amnestic subgroups. A Pearson Chi Squared test was used to compare gender, race, *APOEε4* status and frequency of baseline CDR 0.5 score in the language, typical and memory subgroups. Generalized estimating equations (GEE) [21] were used to model the relationship over time of each subgroup with various outcome variables. GEE take into account multiple visits per participant and that characteristics of the same individual are likely correlated over time. The repeated measures for each participant are treated as a cluster. Outcome variables included MMSE, CDR sum of boxes, each CDR box score, total FAQ and each FAQ category score. Predictor variables included: time (years from baseline), subgroup (dysexecutive, amnestic or typical) and the time _×_ subgroup interaction. The following time stationary covariates were also included in the model: age at first evaluation, education, African American ethnicity and *APOE* genotype. Sex was not included as a covariate because subgroups had similar sex frequencies in our previous analysis [11]. We tested whether the outcome variables at baseline and the rate of change over time of the outcome variables differed in the three subgroups.

## Results

Among the 943 participants, 165 (17.5%) were classified as the dysexecutive subgroup, 157 (16.6%) were classified as the amnestic subgroup and 621 (65.8%) were classified as the typical subgroup. Participant demographic and clinical characteristics are given in Table 1. A full comparison of demographic characteristics and *APOEε4* frequency in the two subgroups is addressed in another publication [11], but we include unadjusted p-values in the table for completeness. Mean years from reported symptom onset to baseline visit, mean years followed and mean number of visits did not differ between subgroups.

**Table 1:** Characteristics of the subgroups.

	dysexecutive subgroup (n = 165)	typical subgroup (n = 621)	amnestic subgroup (n = 157)	p-value
Females (%)	74 (44.8)	308 (49.6)	81 (51.6)	.44
mean age at baseline (SD)	77.1 (8.7)	76.9 (8.2)	75.5 (7.4)	.005
whites (%)	142 (86.1)	543 (87.4)	143 (91.1)	.35
African Americans (%)	13 (7.9)	39 (6.3)	10 (6.4)	.76
mean years of education (SD)	14.3 (3.4)	14.8 (3.1)	15.2 (2.9)	.001
*APOEε4* carriers (%)	98 (59.4)	359 (57.8)	109 (69.4)	.03
mean years followed (SD)	2.3 (1.1)	2.3 (1.1)	2.5 (1.1)	.63
Mean years from reported symptom onset to baseline visit (SD)	5.0 (2.5)	4.9 (2.9)	5.1 (3.2)	.76
mean number of visits (SD)	3.1 (1.0)	3.1 (1.0)	3.2 (1.0)	.48
mean baseline MMSE (SD)	22.6 (3.2)	23.8 (3.2)	24.6 (2.6)	<.001
Frequency of baseline CDR 0.5 (%) (as compared with CDR 1.0)	76 (46.1)	325 (52.3)	81 (51.6)	.36
mean baseline CDR sum of boxes (SD)*	4.4 (1.7)	4.1 (1.7)	4.0 (1.7)	.16
Mean baseline total FAQ (SD)	13.0 (6.9)	12.1 (7.2)	11.9 (6.7)	.32

* 92, 358 and 85 participants in the dysexecutive, typical and amnestic subgroups respectively were analyzed for the CDR sum of boxes analysis.

The mean executive score was −1.03±0.04, −0.06±0.03, and 1.22±0.03 for the amnestic, typical and dysexecutive subgroups respectively. The mean memory score was 1.30±0.06, −0.08±0.03 and −0.93±0.07 for the amnestic, typical and dysexecutive subgroups respectively. Although all participants were required to have memory impairment for a diagnosis of AD, and thus for inclusion in the study, the memory score was significantly different for each of the subgroups (p<.001).

### Comparing Pathologic Diagnosis

Among the 129 autopsy cases, 28 were classified as dysexecutive, 83 were classified as typical and 18 were classified as amnestic. The three subgroups did not differ in odds of having a pathologic diagnosis of AD (Table 2).

**Table 2:** Primary pathologic diagnoses of the subgroups.

pathologic diagnosis	dysexecutive subgroup (n = 28)	typical subgroup (n = 83)	amnestic subgroup (n = 18)
Normal	0	5 (6)	1 (6)
AD	22 (79)	58 (70)	12 (67)
Lewy Body	2 (7)	9 (11)	0
Vascular dementia	0	1 (1)	1 (6)
FTLD	0	3 (4)	1 (6)
Hippocampal sclerosis	3 (11)	2 (2)	3 (17)
Prion	0	0	0
Other	1 (4)	5 (6)	0

There was no association between subgroup and AD pathologic diagnosis (p = .61).

### Comparing Baseline MMSE and Rate of Cognitive Decline on the MMSE Over time

**Figure 1:**
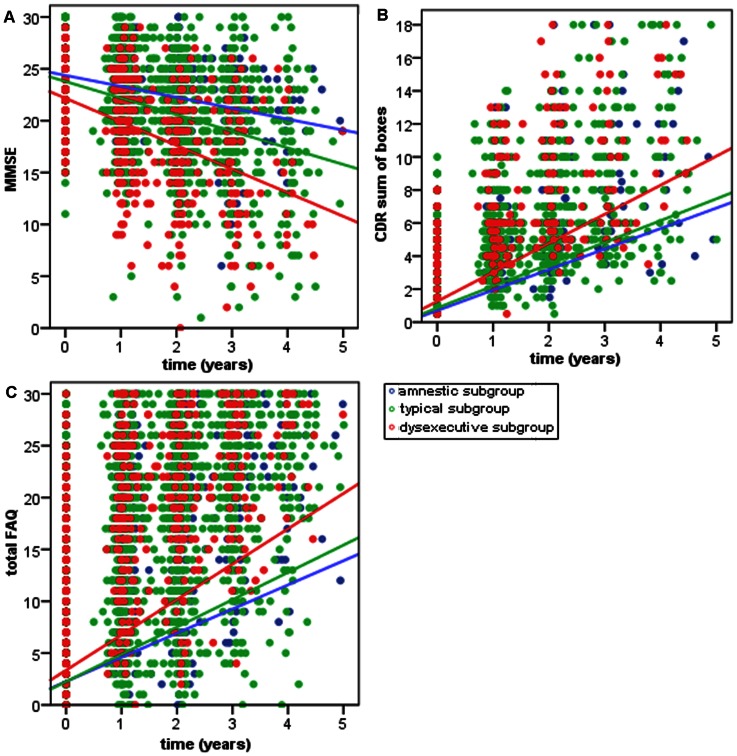
Change in cognition and function over time. Decline in MMSE (A), rise in CDR sum of boxes (B) and (C) rise in total FAQ over time in years in the dysexecutive, typical and amnestic subgroups.

In the GEE model, there were significant main effects for the typical and dysexecutive subgroups indicating that compared with the amnestic subgroup, these subgroups were both more impaired on the MMSE at baseline after controlling for covariates. As expected in AD, all subgroups demonstrated a decrease in MMSE over time, but at different rates. There were significant interaction effects between time and both the typical and dysexecutive subgroups indicating that compared with the amnestic subgroup, the typical and dysexecutive subgroups declined on the MMSE at a faster rate after adjusting for covariates. The typical subgroup declined 52% faster than the amnestic subgroup and the dysexecutive subgroup declined 2.2X (220%) faster than the amnestic subgroup. (Table 3 and figure 1A).

**Table 3:** GEE model with outcome variable MMSE.

effect	b	p
time	−1.06	<.001
typical subgroup	−0.58	.02
dysexecutive subgroup	−2.20	<.001
time × typical subgroup	−0.55	<.001
time × dysexecutive subgroup	−1.22	<.001

b_time_ is the rate of change in MMSE (points/year) for the amnestic subgroup. b_typical_ is the difference in MMSE in the typical subgroup compared with the amnestic subgroup at baseline (time = 0). b_dysexecutive_ is the difference in MMSE in the dysexecutive subgroup compared with the amnestic subgroup at baseline (time = 0). b_time × typical_ is the difference in rate of change in MMSE in the typical subgroup compared with the amnestic subgroup. b_time × dysexecutive_ is the difference in rate of change in MMSE in the dysexecutive subgroup compared with the amnestic subgroup. The following covariates are adjusted for in the model: age at first visit, years of education, *APOEε4* status and African American race.

### Comparing Baseline CDR and Rate of Change in CDR Over Time

In the GEE model, the dysexecutive (but not the typical) subgroup main effect was significant indicating that compared with the amnestic subgroup, the dysexecutive subgroup was more impaired on the CDR sum of boxes at baseline after adjusting for covariates. As expected in AD, all subgroups demonstrated a rise in CDR sum of boxes over time, but at different rates. There was a significant interaction effect between time and the dysexecutive subgroup indicating that compared with the amnestic subgroup, the dysexecutive subgroup demonstrated a faster increase in CDR sum of boxes after adjusting for covariates. The dysexecutive subgroup rose 45% faster than the amnestic subgroup. (Table 4 and figure 1B).

**Table 4:** GEE model with outcome variable CDR sum of boxes.

effect	b	p
time	1.24	<.001
typical subgroup	0.16	.47
dysexecutive subgroup	0.56	.04
time × typical subgroup	0.08	.67
time × dysexecutive subgroup	0.52	.03

b_time_ is the rate of change in CDR sum of boxes (points/year) for the amnestic subgroup. b_typical_ is the difference in CDR sum of boxes in the typical subgroup compared with the amnestic subgroup at baseline (time = 0). b_dysexecutive_ is the difference in CDR sum of boxes in the dysexecutive subgroup compared with the amnestic subgroup at baseline (time = 0). b_time × typical_ is the difference in rate of change in CDR sum of boxes in the typical subgroup compared with the amnestic subgroup. b_time × dysexecutive_ is the difference in rate of change in CDR sum of boxes in the dysexecutive subgroup compared with the amnestic subgroup. The following covariates are adjusted for in the model: age at first visit, years of education, *APOEε4* status and African American race.

When the analysis was repeated using CDR individual box scores, the dysexecutive subgroup was more impaired in the memory, judgment and problem solving and community affairs box scores at baseline compared with the amnestic subgroup. The typical subgroup was more impaired in the judgment and problem solving box score at baseline compared with the amnestic subgroup. Compared with the amnestic subgroup, the dysexecutive subgroup demonstrated a faster rise in box scores for orientation, judgment and problem solving and personal care. (Table S1).

### Comparing Baseline Total FAQ and Rate of Change in Total FAQ Over Time

In the GEE model, the typical and dysexecutive subgroup main effects were not significant indicating that the subgroups did not differ on the total FAQ at baseline after adjusting for covariates. As expected in AD, all subgroups demonstrated a rise in total FAQ over time, but at different rates. There was a significant interaction effect between time and the dysexecutive subgroup indicating that compared with the amnestic subgroup, the dysexecutive subgroup demonstrated a faster increase in total FAQ after adjusting for covariates. The dysexecutive subgroup rose 33% faster than the amnestic subgroup. (Table 5 and figure 1C).

**Table 5:** GEE model with outcome variable Total FAQ.

effect	b	p
time	2.33	<.001
typical subgroup	−0.01	.99
dysexecutive subgroup	1.09	.15
time × typical subgroup	0.30	.23
time × dysexecutive subgroup	0.78	.01

b_time_ is the rate of change in total FAQ (points/year) for the amnestic subgroup. b_typical_ is the difference in total FAQ in the typical subgroup compared with the amnestic subgroup at baseline (time = 0). b_dysexecutive_ is the difference in total FAQ in the dysexecutive subgroup compared with the amnestic subgroup at baseline (time = 0). b_time × typical_ is the difference in rate of change in total FAQ in the typical subgroup compared with the amnestic subgroup. b_time × dysexecutive_ is the difference in rate of change in total FAQ in the dysexecutive subgroup compared with the amnestic subgroup. The following covariates are adjusted for in the model: age at first visit, years of education, *APOEε4* status and African American race.

When the analysis was repeated using individual FAQ categories, at baseline, the dysexecutive subgroup was more impaired in writing checks, paying bills or balancing a checkbook and playing a game of skill or working on a hobby compared with the amnestic subgroup. At baseline, the typical subgroup was more impaired in playing a game of skill or working on a hobby compared with the amnestic subgroup. Compared with the amnestic subgroup, the dysexecutive subgroup demonstrated a faster rise in the following FAQ categories: playing a game of skill or working on a hobby; heating water, making a cup of coffee or turning off the stove; preparing a balanced meal; keeping track of current events; and remembering appointments, family occasions, holidays and medications. Compared with the amnestic subgroup, the typical subgroup demonstrated a faster rise in heating water, making a cup of coffee or turning off the stove. (Table S2).

## Discussion

While AD “classically” presents with episodic memory deficits, in actuality, the presentation can be quite heterogeneous. In this study, we explored MMSE, CDR sum of boxes and total FAQ score at baseline and longitudinally in a dysexecutive subgroup, a typical subgroup and an amnestic subgroup of AD. We restricted our analysis to participants with initially mild AD (CDR≤1) because identification of cognitive subgroups can be challenging later in the disease course.

While subgroup categorization was based on only two neuropsychological tests, the construction of the subgroups is nonetheless meaningful. In this same dataset, we have previously shown that the *APOEε4* allele is underrepresented in the dysexecutive subgroup relative to the amnestic subgroup [11]. This finding is also apparent in table 1 of the current study. In addition, using data from the Alzheimer’s Disease Neuroimaging Initiative (ADNI), investigators have shown that subgroups derived from nearly identical methodology are biologically different, with the dysexecutive subgroup exhibiting greater frontoparietal cortical thinning on MRI than the amnestic subgroup [9].

The amnestic subgroup accounted for about 1/6 of the study sample. While this might seem surprising because most patients with AD have memory deficits, in fact many AD patients also have executive dysfunction [22]. For this reason, a relative measure of executive to memory function was used in this study. The amnestic subgroup does not represent “typical” AD, but rather a focal presentation used to create a clear distinction from the dysexecutive subgroup. Rather, the subgroup that we have termed “typical,” which has relatively equivalent executive and memory dysfunction, is the most common presentation, accounting for about 2/3s of the study sample.

After controlling for covariates, the dysexecutive subgroup had more impairment on the MMSE and CDR sum of boxes than the amnestic subgroup at baseline. This finding, along with the fact that the dysexecutive subgroup was required to have both memory and executive dysfunction, might suggest that participants in the dysexecutive subgroup were not inherently different from participants in the other subgroups, but were merely scoring differently due differences in disease stage. However, there are several reasons why this is unlikely to be the case. The mean time from reported symptom onset to the baseline visit did not differ in the three subgroups. This suggests that more impairment at the baseline visit was due to faster progression from the time of symptom onset. Additionally, even though our sample of participants was required to have memory deficits (but not necessarily executive deficits) as part of the AD diagnostic process, memory was more impaired in the amnestic subgroup than the dysexecutive subgroup. Stated differently, it was not the case that the dysexecutive subgroup had memory deficits comparable to the amnestic subgroup with the added burden of executive dysfunction. Taken together, these findings suggest that the differences in the subgroups are unlikely to be explained by differences in disease stage.

Compared with the amnestic subgroup, the typical and dysexecutive subgroups demonstrated faster decline on the MMSE after adjusting for covariates. On average, members of the dysexecutive subgroup would take about 4 years to undergo a similar amount of cognitive decline as members of the amnestic subgroup would undergo in 9 years. To our knowledge, an analysis of rate of cognitive decline in a dysexecutive subgroup of AD has not been published previously. *APOEε4* status, ethnicity and years of education have all been implicated in the rate of cognitive decline in AD [23]–[26]. In addition, we have shown that *APOEε4* status, age, ethnicity and years of education differ in the dysexecutive and amnestic subgroups at baseline [11]. After including these variables as covariates in our model, differences in rate of decline across the three subgroups persisted, suggesting that the subgroups differ not only in the cognitive phenotype, but in aspects of disease course as well.

Compared with the amnestic subgroup, the dysexecutive subgroup demonstrated a faster increase in CDR sum of boxes and total FAQ after adjusting for covariates. On average, members of the dysexecutive subgroup would take about 5 years to undergo a similar amount of functional decline as members of the amnestic subgroup would undergo in 7 years. This finding more definitively confirms a similar non-significant trend found in ADNI [9]. After standardizing the rate of decline on the MMSE, the CDR sum of boxes and the total FAQ, the difference in rate of decline between the subgroups was about twice as large for cognition as for function. This might be due to the concept that other factors contribute to functional impairment besides cognition. The literature suggests these might include psychiatric features such as depression and apathy [12], [27], although when we included these covariates in our model, the magnitude of our findings did not change (data not shown).

Even though all study participants had a clinical diagnosis of Probable AD, we investigated the possibility that that the dysexecutive subgroup might have disproportionate non-AD pathology (most likely Frontotemporal Lobar Degeneration (FTLD)). However, in the subset of study participants who underwent autopsy, there was no difference in odds of AD pathology across the amnestic, typical and dysexecutive subgroups. Further, out of 129 autopsy cases, there were only 4 cases of FTLD, none of which was a member of the dysexecutive subgroup. These findings suggest that variation in non-AD pathologies across subgroups did not lead to the observed longitudinal differences.

Rather than defining atypical AD subgroups based on cognitive phenotype, Murray et al. defined subgroups based on regional distribution of tau pathology in the brain at autopsy. Compared with a pathologically defined “limbic predominant” AD subgroup, a “hippocampal sparing” AD subgroup more frequently received non-AD clinical diagnoses. The hippocampal sparing subgroup, like our dysexecutive subgroup, demonstrated faster cognitive decline [28]. While comparing burden and anatomic distribution of tau pathology in the autopsy subset of our study would certainly be worthwhile, unfortunately, this data is not available in the NACC dataset.

The study provides additional evidence that a dysexecutive subgroup of AD has distinct characteristics compared with an amnestic subgroup of AD. These differences are not just in phenotype, but also in disease course. Well defined AD clinical phenotypes are important for differential diagnosis and prognostication in clinical practice and in uniform patient recruitment for genetic studies and clinical trials.

## Supporting Information

Table S1 **GEE models with outcome variables CDR box scores.** b_time_ is the rate of change in CDR box score (points/year) for the amnestic subgroup. b_typical_ is the difference in CDR box score in the typical subgroup compared with the amnestic subgroup at baseline (time = 0). b_dysexecutive_ is the difference in CDR box score in the dysexecutive subgroup compared with the amnestic subgroup at baseline (time = 0). b_time × typical_ is the difference in rate of change in CDR box score in the typical subgroup compared with the amnestic subgroup. b_time × dysexecutive_ is the difference in rate of change in CDR box score in the dysexecutive subgroup compared with the amnestic subgroup. The following covariates are adjusted for in the model: age at first visit, years of education, *APOEε4* status and African American race.
(DOCX)

Table S2 **GEE models with outcome variables FAQ categories.** b_time_ is the rate of change in FAQ category score (points/year) for the amnestic subgroup. b_typical_ is the difference in FAQ category score in the typical subgroup compared with the amnestic subgroup at baseline (time = 0). b_dysexecutive_ is the difference in FAQ category score in the dysexecutive subgroup compared with the amnestic subgroup at baseline (time = 0). b_time × typical_ is the difference in rate of change in FAQ category score in the typical subgroup compared with the amnestic subgroup. b_time × dysexecutive_ is the difference in rate of change in FAQ category score in the dysexecutive subgroup compared with the amnestic subgroup. The following covariates are adjusted for in the model: age at first visit, years of education, *APOEε4* status and African American race.
(DOCX)
