# Supplementary material for: Faster Cognitive and Functional Decline in Dysexecutive versus Amnestic Alzheimer's Subgroups: A Longitudinal Analysis of the National Alzheimer's Coordinating Center (NACC) Database
Source: PLoS One. 2013 Jun 3;8(6):e65246. doi: 10.1371/journal.pone.0065246 (PMC3670903; doi:10.1371/journal.pone.0065246)
Supplement: Table S1 — GEE models with outcome variables CDR box scores. btime is the rate of change in CDR box score (points/year) for the amnestic subgroup. btypical is the difference in CDR box score in the typical subgroup compared with the amnestic subgroup at baseline (time = 0). bdysexecutive is the difference in CDR box score in the dysexecutive subgroup compared with the amnestic subgroup at baseline (time = 0). btime × typical is the difference in rate of change in CDR box score in the typical subgroup compared with the amnestic subgroup. btime × dysexecutive is the difference in rate of change in CDR box score in the dysexecutive subgroup compared with the amnestic subgroup. The following covariates are adjusted for in the model: age at first visit, years of education, APOEε4 status and African American race. (DOCX) [file pone.0065246.s001.docx]

Table S1: GEE models with outcome variables CDR box scores

| CDR category | effect | b | p |
| --- | --- | --- | --- |
| Memory | time | 0.21 | <.001 |
|  | typical subgroup | 0.01 | .89 |
|  | dysexecutive subgroup | 0.13 | .03* |
|  | time x typical subgroup | 0.01 | .76 |
|  | time x dysexecutive subgroup | 0.06 | .11 |
| Orientation | time | 0.18 | <.001 |
|  | typical subgroup | -0.06 | .22 |
|  | dysexecutive subgroup | 0.01 | .94 |
|  | time x typical subgroup | 0.04 | .22 |
|  | time x dysexecutive subgroup | 0.10 | .02* |
| Judgment and Problem Solving | time | 0.21 | <.001 |
|  | typical subgroup | 0.07 | .05* |
|  | dysexecutive subgroup | 0.11 | .03* |
|  | time x typical subgroup | -0.004 | .92 |
|  | time x dysexecutive subgroup | 0.12 | .02* |
| Community Affairs | time | 0.21 | <.001 |
|  | typical subgroup | 0.03 | .50 |
|  | dysexecutive subgroup | 0.15 | .007* |
|  | time x typical subgroup | 0.03 | .44 |
|  | time x dysexecutive subgroup | 0.06 | .15 |
| Home and Hobbies | time | 0.25 | <.001 |
|  | typical subgroup | 0.04 | .42 |
|  | dysexecutive subgroup | 0.10 | .14 |
|  | time x typical subgroup | -0.004 | .92 |
|  | time x dysexecutive subgroup | 0.06 | .22 |
| Personal Care | time | 0.19 | <.001 |
|  | typical subgroup | 0.05 | .34 |
|  | dysexecutive subgroup | 0.06 | .36 |
|  | time x typical subgroup | 0.01 | .78 |
|  | time x dysexecutive subgroup | 0.12 | .03* |

b_time_ is the rate of change in CDR box score (points/year) for the amnestic subgroup. b_typical_ is the difference in CDR box score in the typical subgroup compared with the amnestic subgroup at baseline (time = 0). b_dysexecutive_ is the difference in CDR box score in the dysexecutive subgroup compared with the amnestic subgroup at baseline (time = 0). b_time x typical_ is the difference in rate of change in CDR box score in the typical subgroup compared with the amnestic subgroup. b_time x dysexecutive_ is the difference in rate of change in CDR box score in the dysexecutive subgroup compared with the amnestic subgroup. The following covariates are adjusted for in the model: age at first visit, years of education, *APOEε4* status and African American race.
