# Supplementary material for: Faster Cognitive and Functional Decline in Dysexecutive versus Amnestic Alzheimer's Subgroups: A Longitudinal Analysis of the National Alzheimer's Coordinating Center (NACC) Database
Source: PLoS One. 2013 Jun 3;8(6):e65246. doi: 10.1371/journal.pone.0065246 (PMC3670903; doi:10.1371/journal.pone.0065246)
Supplement: Table S2 — GEE models with outcome variables FAQ categories. btime is the rate of change in FAQ category score (points/year) for the amnestic subgroup. btypical is the difference in FAQ category score in the typical subgroup compared with the amnestic subgroup at baseline (time = 0). bdysexecutive is the difference in FAQ category score in the dysexecutive subgroup compared with the amnestic subgroup at baseline (time = 0). btime × typical is the difference in rate of change in FAQ category score in the typical subgroup compared with the amnestic subgroup. btime × dysexecutive is the difference in rate of change in FAQ category score in the dysexecutive subgroup compared with the amnestic subgroup. The following covariates are adjusted for in the model: age at first visit, years of education, APOEε4 status and African American race. (DOCX) [file pone.0065246.s002.docx]

Table S2: GEE models with outcome variables FAQ categories

| FAQ category | effect | b | p |
| --- | --- | --- | --- |
| Writing checks, paying bills or balancing a checkbook | time | 0.21 | <.001 |
|  | typical subgroup | 0.04 | .71 |
|  | dysexecutive subgroup | 0.47 | <.001* |
|  | time x typical subgroup | 0.04 | .37 |
|  | time x dysexecutive subgroup | -0.05 | .36 |
| Assembling tax records, business affairs, or other papers | time | 0.20 | <.001 |
|  | typical subgroup | -0.01 | .90 |
|  | dysexecutive subgroup | 0.21 | .12 |
|  | time x typical subgroup | 0.03 | .48 |
|  | time x dysexecutive subgroup | -0.02 | .77 |
| Shopping alone for clothes, household necessities, or groceries | time | 0.29 | <.001 |
|  | typical subgroup | -0.02 | .82 |
|  | dysexecutive subgroup | 0.09 | .44 |
|  | time x typical subgroup | 0.03 | .43 |
|  | time x dysexecutive subgroup | 0.08 | .08 |
| Playing a game of skill such as bridge or chess, working on a hobby | time | 0.25 | <.001 |
|  | typical subgroup | 0.22 | .002* |
|  | dysexecutive subgroup | 0.21 | .02* |
|  | time x typical subgroup | -0.02 | .57 |
|  | time x dysexecutive subgroup | 0.11 | .02* |
| Heating water, making a cup of coffee, turning off the stove | time | 0.19 | <.001 |
|  | typical subgroup | -0.03 | .72 |
|  | dysexecutive subgroup | -0.07 | .44 |
|  | time x typical subgroup | 0.08 | .05* |
|  | time x dysexecutive subgroup | 0.26 | <.001* |
| Preparing a balanced meal | time | 0.26 | <.001 |
|  | typical subgroup | -0.01 | .96 |
|  | dysexecutive subgroup | -0.002 | .99 |
|  | time x typical subgroup | 0.02 | .73 |
|  | time x dysexecutive subgroup | 0.11 | .04* |
| Keeping track of current events | time | 0.24 | <.001 |
|  | typical subgroup | -0.07 | .34 |
|  | dysexecutive subgroup | -0.01 | .95 |
|  | time x typical subgroup | 0.04 | .24 |
|  | time x dysexecutive subgroup | 0.10 | .02* |
| Paying attention to and understanding a TV program, book, or magazine | time | 0.21 | <.001 |
|  | typical subgroup | -0.06 | .43 |
|  | dysexecutive subgroup | -0.001 | .99 |
|  | time x typical subgroup | 0.03 | .42 |
|  | time x dysexecutive subgroup | 0.08 | .07 |
| Remembering appointments, family occasions, holidays, medications | time | 0.21 | <.001 |
|  | typical subgroup | -0.06 | .42 |
|  | dysexecutive subgroup | 0.05 | .59 |
|  | time x typical subgroup | 0.04 | .17 |
|  | time x dysexecutive subgroup | 0.08 | .04* |
| Traveling out of the neighborhood, driving, or arranging to take public transportation | time | 0.25 | <.001 |
|  | typical subgroup | -0.02 | .83 |
|  | dysexecutive subgroup | 0.16 | .16 |
|  | time x typical subgroup | 0.02 | .60 |
|  | time x dysexecutive subgroup | 0.03 | .49 |

b_time_ is the rate of change in FAQ category score (points/year) for the amnestic subgroup. b_typical_ is the difference in FAQ category score in the typical subgroup compared with the amnestic subgroup at baseline (time = 0). b_dysexecutive_ is the difference in FAQ category score in the dysexecutive subgroup compared with the amnestic subgroup at baseline (time = 0). b_time x typical_ is the difference in rate of change in FAQ category score in the typical subgroup compared with the amnestic subgroup. b_time x dysexecutive_ is the difference in rate of change in FAQ category score in the dysexecutive subgroup compared with the amnestic subgroup. The following covariates are adjusted for in the model: age at first visit, years of education, *APOEε4* status and African American race.
